# Attachment to Group and Mental Health Following the October 7th Attack: The Mediating Role of Meaning in Life and Intolerance of Uncertainty

**DOI:** 10.3390/bs15070879

**Published:** 2025-06-27

**Authors:** Yitshak Alfasi, Avi Besser

**Affiliations:** 1 Department of Behavioral Sciences, Interdisciplinary Faculty for Science, Health and Society, Jerusalem Multidisciplinary College, Jerusalem 91010, Israel; 2 Department of Communication Disorders, Interdisciplinary Faculty for Society and Community, Jerusalem Multidisciplinary College, Jerusalem 91010, Israel

**Keywords:** attachment, attachment to group, mental health, meaning in life, intolerance of uncertainty

## Abstract

Attachment theory, originally developed to explain interpersonal relationships, may also be relevant to understanding the psychological aspects of group belonging. Accordingly, the current study examined the role of attachment to Israel as a group of belonging in shaping mental health outcomes among Israeli citizens following the October 7th attack, focusing on psychological mechanisms involving meaning in life and intolerance of uncertainty. A sample of 1179 participants completed an online survey assessing attachment to Israel, sense of meaning in life, intolerance of uncertainty, and mental health. Path analysis revealed that attachment to Israel was positively associated with better mental health, both directly and indirectly through two key mechanisms: a stronger sense of meaning in life and lower intolerance of uncertainty. Specifically, attachment to Israel was positively associated with meaning in life, which, in turn, was significantly linked to enhanced mental well-being. Moreover, attachment to Israel was associated with lower intolerance of uncertainty, which was also positively correlated with improved mental health. These findings demonstrate that a strong attachment to Israel can enhance mental health by fostering a sense of meaning in life and reducing uncertainty. The implications of these findings are discussed within the frameworks of existential psychology, social identity theory, and attachment theory.

## 1. Introduction

Attachment theory (Bowlby, 1969/1982, 1973, 1980), originally developed to explain interpersonal relationships, may also be relevant to understanding the psychological aspects of group belonging. In times of threat and distress, social groups can serve as attachment figures, providing a sense of security and comfort (Mikulincer & Shaver, 2023). Research (e.g., Greenaway et al., 2016; Jetten et al., 2015; Postmes et al., 2019) suggests that group belonging can alleviate stress and anxiety, enhance self-esteem and perceived competence, and reduce uncertainty. Understanding the connection between group attachment, mental health, and coping mechanisms in stressful situations may provide insights into psychological resilience during collective crises.

Facing war and terrorism takes a toll not only physically but also mentally. Due to ongoing ethno-political conflict, many citizens in the State of Israel may encounter traumatic events throughout their lives, including missile attacks, knife assaults, and suicide bombings. For over two decades, security tensions have persisted between the State of Israel and the Hamas organization, resulting in continuous firing into Israel, particularly around the Gaza Proximity Zone. The Hamas organization’s attacks on 7 October 2023 caused significant distress among citizens, as documented in recent studies across various populations—students (Dopelt & Houminer-Klepar, 2024), parents of children with and without special needs (Alfasi et al., 2025), and the general population (Alfasi & Besser, 2024). This attack not only exacerbated existing mental health challenges but also highlighted the need for effective mental health interventions tailored to diverse populations experiencing terrorism-related trauma. Previous studies conducted in Israel following war and terror events have highlighted the effects of these events on mental health in both the short and long term (Ben Avraham et al., 2022; Besser & Neria, 2010; Besser & Neria, 2012; Besser et al., 2009; Besser & Priel, 2010; Weinberg et al., 2016). In light of these findings, the present study examines the relationship between group attachment and mental health among Israeli citizens after the attacks, focusing on the psychological mechanisms underlying this association. Specifically, it investigates the mediating roles of a sense of meaning in life and intolerance of uncertainty (IU) in this relationship.

Previous research on interpersonal attachment (Alfasi et al., 2025; Bodner et al., 2014; Shaver & Mikulincer, 2012) has demonstrated an association between attachment patterns and meaning in life, with individuals exhibiting secure attachment reporting higher levels of meaning in life, which in turn enhances their psychological well-being. Additionally, IU has been identified as a mediator between attachment patterns and mental health in prior research conducted in Israel during collective crises, including the COVID-19 pandemic (Alfasi, 2022). Based on these findings, the current study will assess whether these variables mediate the proposed link between group attachment and mental health among Israeli citizens in the aftermath of October 7th.

Recent studies on the psychological impact of the war in Ukraine (e.g., Kurapov et al., 2023) have documented significant declines in mental health among Ukrainian civilians, including elevated levels of anxiety, depression, and stress. These findings highlight the broader relevance of the current study’s framework by suggesting that, across different national contexts, attachment to a collective entity—such as one’s country—may serve as a vital psychological resource that promotes resilience in the face of collective trauma.

### 1.1. Literature Review

#### 1.1.1. Attachment to Groups

In infancy and early childhood, primary caregivers—typically one or both parents—function as the main attachment figures. As individuals transition into adolescence and adulthood, this role expands to include siblings, extended family members, close friends, and romantic partners. Additionally, attachment bonds can form with authority figures such as teachers, supervisors, and therapists, as well as within organizational or social groups (Mikulincer & Shaver, 2023). Bowlby (1969/1982) originally proposed that social groups, like individual attachment figures, can provide a sense of security (i.e., act as a “secure base” and a “safe haven”), with attachment bonds extending beyond family members to institutions such as schools, workplaces, religious communities, and political organizations, which may serve as either secondary or primary attachment figures.

This extension of attachment beyond close relationships to larger social groups has deep evolutionary roots. From an evolutionary perspective, social group affiliation was crucial for human survival, enabling early humans to hunt efficiently, defend their communities, and later thrive in agricultural societies (Caporael, 2001). The same instinct that drives an infant to seek proximity to a caregiver is reflected in individuals’ attachment to social groups. Like caregivers, groups offer protection, stability, and a secure base for exploration and personal development (Sochos, 2014).

Several factors highlight the role of social groups as a “secure base”, similar to human attachment figures (Mikulincer & Shaver, 2023). Just as infants seek comfort from caregivers, individuals turn to their groups for reassurance and protection. Caregivers provide emotional support, just as social groups foster emotional stability among their members. Additionally, identity formation occurs both through caregiver relationships and group affiliations, shaping self-perception and belonging. While infants initially depend on caregivers before developing independence, group members receive support while gradually cultivating autonomy. Thus, attachment to a group serves as both a protective mechanism and a foundation for personal growth and mental well-being.

This regulatory role of group attachment differs conceptually from other group-related constructs such as social identification and identity fusion. While social identification involves a sense of belonging and cognitive alignment with the group (Daniel et al., 2016), and identity fusion reflects a symbolic merging of the personal and collective self (Fredman et al., 2017), group attachment emphasizes the regulatory function of the group as a secure base. It is not about identity centrality or symbolic overlap, but about whether the group provides psychological anchoring in the face of threat.

Terror Management Theory (TMT) provides empirical support for the psychological role of group attachment, particularly in times of danger and uncertainty. Castano and Dechesne (2005) demonstrated that reminders of mortality increase social identification and perceptions of group entitativity, reinforcing the notion that group attachment provides continuity and emotional security, much like interpersonal attachment. Fritsche et al. (2008) further showed that mortality salience enhances support for ingroups and cultural norms, a process they interpret as an attempt to restore a sense of control. Notably, their findings indicate that only pure mortality salience—not other types of threats—elicits strong group-protective responses, suggesting that group attachment is not merely a defense against existential anxiety but also a psychological strategy for maintaining control and stability during crises.

Beyond existential threat management, research on social identification and self-esteem highlights additional benefits of group attachment. Jetten et al. (2015) found that belonging to multiple meaningful social groups enhances self-esteem, as group membership provides psychological resources, a sense of meaning, and pride. Across multiple studies, individuals with greater group affiliations reported higher self-esteem, with collective self-esteem mediating this effect rather than simple social connections. These findings suggest that group attachment serves as a psychological buffer, fostering resilience and well-being beyond mortality-related concerns.

The meta-analysis by Postmes et al. (2019) further underscores the link between group attachment and mental health, particularly in relation to depression. Analyzing 76 studies (N = 31,016), they found that stronger social identification was associated with lower depression levels, though this effect varied depending on the type of group. The association was stronger in interactive groups (e.g., family, close friends, workplace teams), where direct interpersonal interaction occurs, whereas categorical groups (e.g., nationality, religion, political affiliation) showed a more moderate effect. However, within the national identity category, stronger identification with one’s nation was linked to lower depression levels, particularly when national identity was perceived as a source of meaning, stability, and security (Greenaway et al., 2016; Sani et al., 2012). These findings indicate that identification with large, abstract social groups can help reduce depression, even when this effect cannot be directly attributed to social capital or interpersonal support. This aligns with Haslam et al.’s (2009) argument that social identification provides psychological benefits beyond interpersonal support, particularly by fostering meaning-making and fulfilling psychological needs.

The psychological benefits of group attachment become particularly relevant in times of crisis, when individuals seek stability and coping mechanisms to manage distress. In this context, a recent study conducted in Israel several weeks after the October 7th attacks found that attachment to Israel was positively associated with mental health (Alfasi & Besser, 2024). Notably, this relationship was mediated by adaptive emotion regulation strategies, particularly task-focused coping, which promotes problem-solving and resilience, rather than emotion-focused coping, which tends to amplify negative emotions. These findings further support the idea that group attachment functions not only as an existential buffer but also as a psychological mechanism that enhances well-being by promoting adaptive coping strategies during adversity. Furthermore, understanding how different socio-economic and cultural contexts shape these responses can illuminate potential disparities in mental health outcomes across global populations affected by terrorism.

#### 1.1.2. Meaning in Life

The pursuit of meaning in life is an important buffer against distress, particularly during challenging times (Frankl, 1959/1992). Meaning in life is recognized as a positive psychological quality that contributes to overall well-being and serves as a cornerstone for mental health (He et al., 2023; Steger & Dik, 2009; Steger & Frazier, 2005). Individuals with a strong sense of meaning in life are generally better equipped to cope with stressors and psychological burdens, which is especially vital during periods of crisis, such as the aftermath of the October 7th attacks. Frankl (1959/1992) emphasized that the absence of meaning in life can lead to poor functioning and psychological symptoms, including depression, anxiety, suicidal behavior, and dependency (Debats, 1996; Mascaro & Rosen, 2006). This suggests that a lack of meaning in life may intensify emotional turmoil during collective trauma, further underscoring the role of meaning in life as a protective factor against mental health challenges.

Research on interpersonal attachment indicates that attachment patterns are associated with the sense of meaning in life, as securely attached individuals are more likely to perceive their lives as meaningful (Bodner et al., 2014; Shaver & Mikulincer, 2012). Individuals with secure attachment benefit from a sense of psychological safety (i.e., a “secure base”), allowing them to redirect mental resources away from anxiety regulation toward exploration, learning, and personal development. This shift fosters a greater capacity to find purpose and meaning in different aspects of life. Furthermore, secure attachment is linked to strong and lasting interpersonal relationships, which contribute to a deep sense of belonging, a fundamental aspect of meaning in life (Krause, 2007; Lambert et al., 2013).

On the other hand, individuals with insecure attachment are more likely to struggle with feelings of meaninglessness. Those with high attachment anxiety, often characterized by low self-confidence and diminished self-worth (Mikulincer & Shaver, 2007), may find it challenging to recognize value and significance in their actions across various life domains. Additionally, their difficulties in maintaining close and meaningful relationships over time disrupt their sense of belonging, which is central to experiencing meaning in life. Similarly, individuals with high attachment avoidance frequently view others negatively and experience emotional detachment and isolation (Mikulincer & Shaver, 2007), which restricts their ability to derive meaning from interpersonal connections. More broadly, their skeptical and sometimes cynical worldview makes it harder for them to see meaning and value in their actions beyond their immediate utility.

Following traumatic events, individuals may experience an increased need to find meaning in their experiences to regulate the heightened anxiety they feel. In such situations, a sense of meaning in life can serve as a psychological resilience mechanism, facilitating better coping and enhanced recovery. Supporting this notion, Alfasi and Besser (2024) conducted a study in Israel following the October 7th attacks, which found that individuals with secure attachment reported a stronger sense of meaning in life, which in turn mitigated the psychological distress they experienced as a result of the events. In contrast, individuals with high levels of attachment anxiety or avoidance reported lower levels of meaning in life, which corresponded with greater psychological distress.

#### 1.1.3. Intolerance of Uncertainty

Intolerance of uncertainty (IU) is a psychological construct referring to the negative emotional responses that individuals experience when faced with ambiguous situations (Carleton et al., 2012). IU has been defined as “the tendency of an individual to consider the possibility of a negative event occurring as unacceptable, irrespective of the probability of occurrence” p. 105 (Carleton et al., 2007). Additionally, it involves “an individual’s dispositional incapacity to endure the aversive response triggered by the perceived absence of salient, key, or sufficient information, and sustained by the associated perception of uncertainty” p. 31 (Carleton et al., 2012). Research indicates that IU plays a central role in the etiology and maintenance of worry and rumination, explaining its transdiagnostic associations with various psychological disorders (Yook et al., 2010).

Previous research has demonstrated a link between interpersonal attachment patterns and levels of tolerance for uncertainty. Specifically, secure attachment is associated with greater tolerance for uncertainty and more adaptive coping strategies, whereas insecure attachment is often linked to heightened IU and maladaptive behaviors (Boelen et al., 2014; Nekic & Mamic, 2019). Individuals with secure attachment tend to be better equipped to navigate unpredictable situations, employing flexible coping strategies, such as seeking support from peers or constructively processing ambiguous information. In contrast, those with insecure attachment are more prone to excessive worry, often relying on avoidance or compulsive behaviors, which further exacerbate their IU and negatively affect their mental health. This highlights a key mechanism through which attachment influences mental health, reinforcing emotional resilience while improving individuals’ ability to manage uncertainty.

Tolerance for uncertainty becomes particularly significant in times of crisis, such as pandemics, terrorist attacks, and war (Alfasi et al., 2025; Davenport et al., 2020). During such events, the tendency to perceive uncertainty as a threat intensifies, often driving individuals toward maladaptive coping mechanisms in an attempt to regain a sense of control. For instance, those with higher IU may compulsively seek information or avoid uncertain situations altogether, reinforcing their anxiety rather than alleviating it.

Accordingly, IU has been consistently linked to poor mental health outcomes during global crises, such as the COVID-19 pandemic (Rettie & Daniels, 2021). A study conducted in Israel across two phases of the pandemic found that IU mediated the relationship between attachment anxiety and both emotional well-being and stress (Alfasi, 2022). Likewise, research on parents of children with and without special needs following the October 7th attacks revealed that IU mediated the association between attachment anxiety and both mental health and parental stress across both groups (Alfasi et al., 2025).

#### 1.1.4. The Current Study: Overview and Hypotheses

As reviewed thus far, the literature highlights the significance of group attachment in shaping psychological resilience, particularly in times of crisis. Accordingly, the current study aims to examine the relationship between group attachment and mental health among Israeli citizens following the October 7th attacks, as well as the psychological mechanisms underlying this association.

In the present study, group attachment is conceptualized as attachment to Israel. In this sense, attachment to Israel as a group of belonging functions not only as an interpersonal bond but also as an identity-based attachment, rooted in collective identity, shared history, and symbolic meaning. Prentice et al. (1994) distinguish between “common bond” attachment, which arises from affection and emotional ties among group members, and “common identity” attachment, which is shaped by identification with the group’s values and identity. This distinction aligns with V. D. Volkan’s (1999) and V. K. Volkan’s (2013) concept of large-group identity, which describes a shared psychological experience uniting thousands or millions of individuals through a sense of continuity, common history, and symbolic meaning. Unlike small-group attachments, which are based on direct personal interactions, large-group identity is maintained through collective narratives, rituals, and symbols, providing a stable sense of belonging, particularly in times of crisis.

While group attachment has traditionally been conceptualized as comprising two dimensions—*anxiety* and *avoidance* (Smith et al., 1999)—this framework was developed in the context of small and relationally dense groups (e.g., task-oriented teams, workgroups, therapeutic groups), where members typically know each other personally and interact in ways similar to close relationships. However, the current study examines attachment to a large symbolic group—the nation—which differs in its impersonal and imagined nature. As suggested by V. D. Volkan (1999), large-group identity is rooted not in interpersonal familiarity, but in shared history, symbols, and collective narratives. In such contexts, group attachment is less about interpersonal regulation and more about whether the collective is perceived as a psychological anchor—a source of emotional security and existential coherence in times of threat.

Building on this conceptual distinction, we focus on a unidimensional construct of attachment security, capturing the extent to which individuals perceive Israel as a secure base. This approach aligns with prior work on symbolic group attachment (e.g., Boccato & Capozza, 2011; DeMarco & Newheiser, 2019) and reflects recent efforts to adapt attachment theory to broader social and ideological affiliations. Complementing this perspective, previous research (e.g., Castano & Dechesne, 2005; Fritsche et al., 2008) has shown that individuals can also rely on attachment to abstract groups—such as nations, religions, and political organizations—or even to abstract concepts like God (Kirkpatrick, 1999), as a psychological anchor. In attachment theory terms, such entities can serve as a *secure base* that helps regulate anxiety and distress during periods of heightened threat.

The underlying psychological mechanism in this process is that attachment to an abstract group fosters a sense of meaning in life, which in turn mobilizes the mental resources needed to restore a sense of control and reduce uncertainty. When individuals perceive their experiences as meaningful, they are better able to tolerate seemingly random and senseless events, which would otherwise undermine their sense of security and psychological stability.

Accordingly, the present study examines the following hypotheses:

**H1.**  *Attachment to Israel will be positively associated with mental health among Israeli citizens following the October 7th attacks.*

**H2.**  *The relationship between attachment to Israel and mental health will be mediated by a sense of meaning in life.*

**H3.**  *The relationship between attachment to Israel and mental health will be mediated by levels of intolerance of uncertainty.*

**Figure 1:**
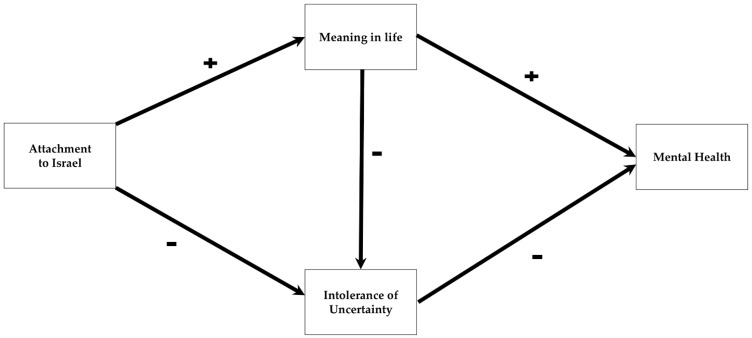
The proposed mediational model illustrating how attachment to Israel is associated with mental health following the October 7th attack, with mediation by meaning in life and intolerance of uncertainty.

Figure 1 presents the proposed model examined in the current study, illustrating the relationship between attachment to Israel and mental health, along with its mediating factors.

## 2. Method

### 2.1. Participants and Procedure

The study took place during December 2024 and included 1214 Jewish-Israeli participants. We excluded data from 35 participants due to them being univariate outliers (*n* = 29), providing inconsistent responses (*n* = 1), or having invariant response patterns (*n* = 5). The final 1179 participants (53.4% women, 46.6% men; M age = 36.86, SD = 7.79, Range: 20–51) fully responded to our survey. The geographic distribution of the participants was as follows: North (21%), South (13%), Central (39%), Jerusalem and surrounding areas (17%), and the Coastal Plain (10%). Religiosity levels were distributed as follows: 5.7% identified as atheists, 41.1% as secular, 17.6% as traditional, 15% as religious, and 21% as ultra-Orthodox. In terms of education levels, 35.8% had completed secondary education, 39.9% held a bachelor’s degree, 22.6% had a master’s degree or higher, and 1.6% held a Ph.D. Employment data indicated that 88% of participants were employed. Monthly gross household income was reported as below the national workforce average by 35.1% of participants, at the average by 37.2%, and above average by 27.7%.

Participants provided information on political stance, rated on a 1 (left-wing) to 7 (right-wing) scale. The majority of the sample (68.8%) scored above 4, indicating a right-wing orientation (M = 5.22, SD = 1.48), which reflects the political landscape in Israel. Participants also reported cumulative exposure to traumatic events related to the October 7th attacks through various channels, including media and social network exposure, knowing someone who was injured, kidnapped, or killed, having a family member who was harmed, personal involvement (as a soldier or attendee at the Nova festival), experiencing an attack on their village, spending time in a security/shelter room, or being evacuated from their home. Overall, 88.5% of participants experienced 1 to 3 such events (M = 2.21, SD = 1.09).

Participants were recruited through ‘iPanel,’ a local online panel where individuals register to take part in studies in exchange for monetary compensation. In this study, they received 10 ILS (approximately USD 2.5) for their participation. Respondents completed an online questionnaire via a secure platform, providing informed consent before answering demographic questions and measures assessing mental health, attachment to Israel, meaning in life, and intolerance of uncertainty. They were informed that they could withdraw at any time, and no identifying information was obtained. The study was approved by the Ethics Committee of the Jerusalem Multidisciplinary College (IRB protocol code #568). The data file is publicly available on the Open Science Framework (OSF) at: https://osf.io/8sefx/.

### 2.2. Measures

#### 2.2.1. Attachment to Israel

Group attachment to Israel was assessed using a scale developed by Alfasi and Besser (2024), which was adapted from Smith et al.’s (1999) Social Group Attachment Scale. Participants were asked to rate their agreement with eight statements reflecting their attachment to Israel on a 7-point Likert scale, ranging from 1 (*strongly disagree*) to 7 (*strongly agree*). For example: “I feel comfortable being dependent on Israel”; “I find it difficult to trust Israel completely or to be dependent on Israel” (*reversed item*); and “I know Israel will be there when I need it.” Alfasi and Besser (2024) reported high internal consistency for the scale and demonstrated that all items loaded onto a single factor, supporting its unidimensional structure. In the current study, a principal component analysis with an unrotated solution also indicated a clear single-factor structure, with an eigenvalue of 4.29, accounting for 53.66% of the total variance. In addition, the scale showed high internal consistency, with a Cronbach’s alpha of 0.87.

#### 2.2.2. Meaning in Life

Meaning in life was assessed using the Meaning in Life Questionnaire (MLQ; Steger et al., 2006), which evaluates individuals’ perceptions of meaning in their lives through two dimensions: presence of meaning (e.g., “I understand the meaning of my life”) and search for meaning (e.g., “I am looking for something that makes my life feel meaningful”). Participants rated their agreement with 10 statements (five for each dimension) on a 7-point Likert scale ranging from 1 (strongly disagree) to 7 (strongly agree). In the present study, only the “presence of meaning” subscale was measured, as it reflects psychological stability and coherence, in line with the study’s focus on protective factors in the aftermath of trauma. The “search for meaning” subscale, which often captures existential doubt or distress, was excluded. Higher scores on the presence subscale reflect a stronger sense of life meaning (α = 0.88).

#### 2.2.3. Intolerance of Uncertainty

Intolerance of uncertainty was assessed using the Intolerance of Uncertainty Scale (IUS-12; Carleton et al., 2007), which measures responses to uncertainty and ambiguous situations, as well as the extent to which uncertainty is perceived as stressful and disabling (e.g., “Unexpected events upset me greatly” and “Uncertainty prevents me from living a full life”). Items were scored on a 7-point Likert scale ranging from 1 (*strongly disagree*) to 7 (*strongly agree*), with higher scores indicating greater intolerance of uncertainty (α = 0.91).

#### 2.2.4. Mental Health

Mental health was measured using the Mental Health Inventory (MHI-5) (Berwick et al., 1991). This scale assesses mental health status through a five-item screening test that evaluates feelings of anxiety, depression, positive affect, and behavioral/emotional control. Participants were asked to indicate the extent to which they had felt the following way during the last few weeks since the war broke out, using a scale from 1 (*very little*) to 7 (*very much*): ‘nervous and tense’ (anxiety), ‘calm and peaceful’ (positive affect), ‘discouraged and depressed’ (depression), ‘happy and joyful’ (positive affect), ‘so down in the dumps that nothing could cheer you up’ (behavioral/emotional control). Items were coded so that higher scores reflect greater mental health (α = 0.87).

## 3. Results

### 3.1. Data Analysis Strategy

Data were analyzed using SPSS version 26 (SPSS Inc., Chicago, IL, USA). First, Pearson bivariate correlation tests were conducted to examine the associations among the study variables. Next, path analyses were performed in AMOS (Version 29; Arbuckle, 2023) using the maximum-likelihood method to test total, direct, and indirect (mediational) effects. We chose path analysis as our primary method because it allows for the simultaneous examination of multiple mediators and complex relationships within the proposed model, providing a comprehensive view of the direct and indirect effects.

Path coefficients were estimated using 5000 bootstrap samples, with all samples (100%) successfully converging. We used bootstrapping, a resampling technique involving repeated sampling with replacement, to estimate the confidence intervals of our indirect effects. This approach helps to assess the stability of the estimates without relying on the assumption of normality and provides valuable information about the likely range of the parameters. In the present study the 95% confidence intervals and percentile bootstrap confidence intervals for the estimated parameters and indirect effects confirmed that the indirect effects were significantly different from zero, providing a stable estimate of the distributions. All statistical tests were conducted using two-tailed significance tests, with confidence intervals set at *p* < 0.05. Preliminary analyses indicated that controlling for gender and age did not alter the main study findings. Therefore, these variables were excluded from subsequent analyses to maintain clarity and conciseness. Among the demographic variables that could influence the main study variables—exposure to the October 7th events and political orientation—we found that exposure was significantly correlated only with mental health (r = −0.15, *p* < 0.001). In contrast, political orientation was significantly correlated with multiple variables: mental health (r = −0.22, *p* < 0.001), meaning in life (r = −0.24, *p* < 0.001), and attachment to Israel (r = −0.31, *p* < 0.001). These findings suggest that exposure to the events was associated with worse mental health and that leaning towards the right end (coded as 1) of the political orientation spectrum is associated with better mental health, higher meaning in life, and higher attachment to Israel. Therefore, in the final stage of the analysis, we will control for these effects to assess their potential influence on the final model.

### 3.2. Univariate Analyses

Table 1 shows the correlation coefficients and descriptive statistics. The values of skewness and kurtosis indicate that the data are fairly symmetrical (Byrne, 2010; George, 2011; Hair et al., 2010). As shown in Table 1, attachment to Israel is significantly associated with higher levels of mental health and lower levels of intolerance of uncertainty. Meaning in life is also significantly linked to improved mental health and reduced intolerance of uncertainty. Lastly, higher levels of intolerance of uncertainty are significantly associated with lower mental health outcomes.

We examined if there was multicollinearity among the study variables. Eigenvalues of the scaled and uncentered cross-products matrix, condition indices, variance decomposition proportions, variance inflation factors (VIF), and tolerances from the multicollinearity analyses indicated no multicollinearity. VIF measures how much the variance of a regression coefficient is inflated due to multicollinearity among predictors. Values exceeding 5 (or 10, depending on the source) suggest problematic multicollinearity (in the present study, we obtained VIFs < 1.08). Tolerance is the reciprocal of VIF (tolerance = 1/VIF) and indicates the proportion of variance not explained by other predictors. Tolerance values below 0.1 (or 0.2) typically suggest multicollinearity issues (in the present study, we obtained tolerance values in the range of 0.93 to 0.96). To assess the potential impact of common method bias, we conducted Harman’s single-factor test using all items from the four questionnaires in our analysis. This method examines the variance explained by one factor using Principal Axis Factoring as the extraction method. The unrotated factor solution indicated that common method bias is not likely to be a significant issue in this study (Podsakoff et al., 2003); the total variance extracted by one factor was 25.967%, well below the accepted threshold of 50% (MacKinnon et al., 2007). In our analysis, the VIF values were below the recommended thresholds, tolerances were above the critical value, and the first factor explained 26% of the variance, which is below the 50% threshold, indicating that multicollinearity was not problematic and the data passed the Harman’s test.

### 3.3. Multivariate Analyses

The results of the direct and indirect effects path mediation analysis are presented in Table 2.

**Figure 2:**
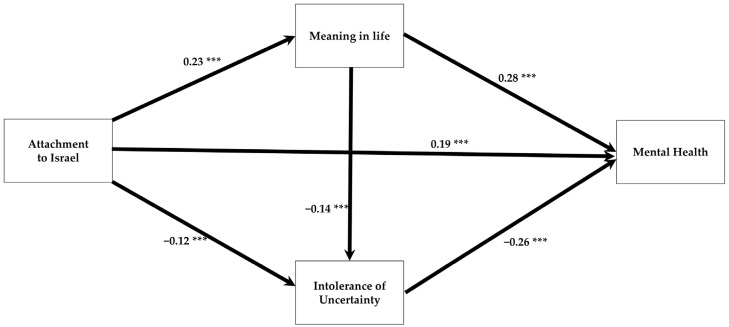
Direct and indirect effect of attachment to Israel on mental health. *** *p* < 0.001.

In accordance with our main hypothesis (H1), Table 2 shows a significant positive total effect of attachment to Israel on mental health (β = 0.29, t = −4.96, CI95% [0.235, 0.355], *p* < 0.0001). Supporting Hypotheses 2 and 3, this association decreased when the mediators—meaning in life and intolerance of uncertainty—were included in the model (β = 0.19, t = 7.14, CI95% [0.135, 0.249], *p* < 0.0001), although the direct effect remained significant. As shown in Table 2, attachment to Israel has both direct and significant indirect positive effects on mental health. It is positively associated with meaning in life, which is significantly linked to improved mental health (H2). Additionally, attachment to Israel is significantly associated with lower intolerance of uncertainty, which in turn correlates with better mental health (H3). Finally, meaning in life was associated with lower intolerance of uncertainty, which is also linked to improved mental health outcomes (see Figure 2).

Finally, following preliminary analyses, we expanded the model presented in Figure 2 to include the effects of exposure to the October 7th events on mental health, as well as the effects of political orientation on mental health, meaning in life, and attachment to Israel. We controlled for the shared variance between exposure and political orientation. The results indicated that the findings described in Figure 2 remained unchanged, and the expanded model fit the data well (χ^2^(df = 4) = 9.47, *p* = 0.05, (χ^2^/df = 2.37), GFI = 1.0; AGFI = 0.99; CFI = 0.99; RMSEA = 0.03–0.06).

## 4. Discussion

This study explored the complex interplay between attachment to Israel, meaning in life, and intolerance of uncertainty (IU) in shaping mental health outcomes following the 7 October 2023, attacks. Our findings underscore the significant positive association between attachment to Israel and mental well-being, reinforcing prior research on the protective role of group attachment in times of crisis (Alfasi et al., 2025; Alfasi & Besser, 2024; Mikulincer & Shaver, 2023).

As predicted, the effect of attachment to Israel on mental health was mediated by a sense of meaning in life. This finding highlights the role of group attachment in fostering a sense of purpose, thereby enhancing psychological resilience. This aligns with existential psychology perspectives, which emphasize the buffering effect of meaning in life against distress (Frankl, 1959/1992; He et al., 2023).

Furthermore, as anticipated, attachment to Israel was associated with lower levels of intolerance of uncertainty. This suggests that strong group attachment provides a sense of security and control, mitigating anxiety and uncertainty during crises. These results align with prior research indicating that secure interpersonal attachment enhances tolerance of uncertainty and promotes adaptive coping strategies (Boelen et al., 2014; Nekic & Mamic, 2019), whereas insecure attachment is linked to heightened IU and maladaptive coping mechanisms.

Notably, the significant direct effect of attachment to Israel on mental health remained even after accounting for mediators, suggesting that group attachment itself—beyond its impact on meaning and IU—offers substantial psychological support. This may stem from stronger social support networks, shared values, and a reinforced sense of belonging, all of which are amplified during crises (Mikulincer & Shaver, 2023). These findings contribute to a deeper understanding of how group attachment functions as a psychological resource, offering both existential and emotional stability in the face of adversity.

The findings of the current study also align with attachment theory, suggesting that individuals’ psychological ties to their group affiliations play a pivotal role in their ability to manage stress and uncertainty. In the first volume of his attachment trilogy, Bowlby (1969/1982) suggested that during times of distress, individuals may turn not only to close relationship partners for support and protection but also to informal social groups and structured organizations:

During adolescence and adult life a measure of attachment behavior is commonly directed not only towards persons outside the family but also towards groups and institutions other than the family. A school or college, a work group, a religious group or a political group can come to constitute for many people a subordinate attachment “figure,” and for some people a principal attachment “figure.” (p. 207)

Conceptualizing groups as attachment figures implies that they can fulfill similar functions to personal attachment relationships, offering security and comfort during distressing times. The physical or symbolic presence of a group can alleviate stress, as members perceive themselves as protected. Moreover, groups can encourage exploration, learning, and goal achievement, paralleling the dynamics observed in close interpersonal attachment relationships. Individuals who strongly identify with their group are more likely to experience these psychological benefits, reinforcing the role of group attachment in emotional resilience (Mikulincer & Shaver, 2023).

However, mere group membership is insufficient to generate these positive psychological effects. Just as the mere presence of a romantic partner does not automatically provide security, the impact of group attachment depends on two key factors. First, the emotional bond between members and their reliance on the group for protection and support. The group must be perceived as a trusted attachment figure that members can turn to in times of distress. Second, the perception that the group is accepting, validating, and supportive of its members’ needs and aspirations. Individuals with stronger emotional ties to the group (i.e., higher group identification) are more likely to benefit from its protective and empowering effects (Mikulincer & Shaver, 2023).

Moreover, groups with a strong shared identity tend to remain cohesive over time, as their existence is not solely dependent on personal relationships between members. Even if interpersonal connections weaken, individuals may still choose to remain in the group due to the sense of identity it provides. This phenomenon is evident in historical social movements, where people remain committed not merely to the movement’s goals but to the collective identity it fosters (V. D. Volkan, 1999).

Additionally, group cohesion plays a significant role in determining whether a group functions as a secure base. Higher group cohesion enhances feelings of safety and belonging among members. However, not all highly cohesive groups promote personal growth and independent exploration. Some tightly knit groups may suppress individuality, discouraging independent thought and personal development. When a group fails to support its members’ personal growth and autonomy, it ceases to function as a true secure base, reducing the likelihood of individual flourishing (Eisenberger & Stinglhamber, 2011).

In extreme cases, highly cohesive groups may adopt controlling mechanisms, where leadership relies on fear, threats, or coercion to maintain power. These groups create an illusion of security, but in reality, they restrict personal growth and limit members’ autonomy. Such groups contrast sharply with healthy, well-functioning groups, which simultaneously provide security and encourage individual expression. The ideal group serves as both a protective environment and a space for personal development, allowing members to feel secure while also fostering independence and self-discovery.

The ability to perceive Israel as a secure base plays a significant role in managing psychological distress. It contributes to maintaining a sense of optimism and hope even during crises. Periods of national distress often see an increase in social solidarity, reinforcing the perception of the group as a source of security (Castano & Dechesne, 2005). In such times, the group becomes a focal point for seeking closeness and comfort, functioning as a safe haven for its members. The challenge, however, is to sustain this perception even in times of stability, ensuring that group cohesion remains strong and that individuals continue to experience their national or social identity as a source of psychological resilience.

In addition to the primary variables of interest, our analyses revealed that exposure to the October 7th events was associated with worse mental health, highlighting the detrimental impact of direct trauma or threat. Conversely, leaning towards the right end (coded as 1) of the political orientation spectrum was linked to better mental health, as well as higher levels of meaning in life and attachment to Israel. This indicates that certain political attitudes may serve as a psychological resource or buffer during times of crisis, possibly reflecting a stronger sense of collective identity or security among those with such orientations. Understanding these associations underscores the importance of considering individual contextual factors, such as trauma exposure and political ideology, in assessing psychological resilience and designing intervention strategies during collective emergencies.

### 4.1. Limitations and Future Directions

While this study benefits from a large sample size and the use of validated psychometric instruments, several limitations should be acknowledged. First, future research could benefit from more precise data regarding participants’ exact places of residence to better assess proximity to the scenes of the attack. Moreover, the cross-sectional design precludes causal inferences, highlighting the need for longitudinal studies to track changes in group attachment and mental health over time. Additionally, the reliance on self-report measures necessitates caution in interpreting the findings. Although common method bias was addressed statistically, future research could benefit from using multiple assessment methods to further mitigate this risk.

Another limitation concerns the absence of pre-attack baseline data, which prevents us from determining whether the level of attachment to Israel changed as a result of the October 7th attack. It is possible that the event led to an increase in ingroup solidarity, thereby enhancing attachment. However, we are able to compare the current findings to data collected immediately after October 7th, in November 2023 (Alfasi & Besser, 2024). In that study, the mean level of attachment to Israel was 4.25, which is quite similar to the 4.03 observed in the present study. This suggests that even if there was a modest increase in group attachment immediately following the attack, it was relatively moderate, and overall group attachment levels remained stable even as the initial emotional impact began to fade. Nonetheless, future longitudinal research could help assess potential changes in group attachment levels over time.

The study did not assess participants’ perceptions of Israel’s external image or the psychological impact of international criticism during the war in Gaza. Given evidence that the benefits of group identification may depend on a group’s perceived social legitimacy, future research should examine how external delegitimization or stigma moderates the psychological effects of national attachment.

Another limitation concerns the conceptual openness of the term “Israel” in the group attachment measure. We intentionally left the term undefined, allowing participants to interpret “Israel” according to their personal, cultural, or ideological frameworks—whether as a state, a historical homeland, a people, or a symbolic collective. This reflects the complex and uniquely layered nature of Israeli national identity, in which Judaism functions simultaneously as a religion and a national affiliation. Such complexity may introduce interpretive variability. Future qualitative or mixed-method research could explore how individuals subjectively define “Israel” in different contexts.

This study opens several avenues for future research. First, longitudinal designs could establish causal relationships by tracking psychological change over time. Second, cross-cultural comparisons of collective trauma responses in societies with varying levels of national cohesion could yield valuable insights. Third, experimental studies manipulating attachment-related cues or meaning-oriented interventions could clarify underlying mechanisms. Additionally, qualitative research could explore how attachment to Israel shapes individuals’ coping strategies, social support experiences, and perceptions of self and community during crises.

Further research should also investigate the specific components of the direct effect of attachment to Israel on mental health—such as increased belonging, perceived social support, or a heightened sense of collective agency. Moreover, future studies should examine how socioeconomic status and cultural background influence the role of group attachment as a resilience factor, in order to address global disparities in mental health outcomes related to terrorism and national threat.

### 4.2. Implications

The findings suggest that fostering group attachment may serve as a practical intervention strategy to enhance psychological resilience among individuals affected by terrorism. This could inform policymakers aiming to implement mental health support systems tailored to the needs of diverse communities, recognizing that resilience factors may vary based on cultural and socio-economic backgrounds.

### 4.3. Conclusions

The current study provides compelling evidence for the role of attachment to social groups through shared identity in fostering mental well-being amid collective trauma. Specifically, it demonstrates that the association between attachment to Israel and mental health is mediated by two key psychological mechanisms: a stronger sense of meaning in life and lower intolerance of uncertainty. These findings offer empirical support for the theoretical assertion that groups can function as attachment figures, much like interpersonal relationships, by fulfilling core psychological needs such as security, stability, meaning, and belonging.

These elements allow individuals to mobilize mental resources for exploration and growth during times of stability, while facilitating more focused and effective coping mechanisms in response to perceived threats and distress during crises. By serving as a psychological anchor—a “secure base”—group attachment plays a crucial role not only in resilience-building but also in activating adaptive mediated pathways that support psychological well-being. In light of global disparities in responses to terrorism, our study underlines the importance of incorporating localized mental health strategies that reflect the cultural contexts of affected populations, ensuring more effective mental health support systems in diverse settings.

## Figures and Tables

**Table 1:** Intercorrelations and descriptive statistics (N = 1179).

	1	2	3	4
1. Attachment to Israel	–			
2. Meaning in Life	0.23 ***	–		
3. Intolerance of Uncertainty	−0.15 ***	−0.17 ***	–	
4. Mental Health	0.29 ***	0.36 ***	−0.33 ***	–
*Mean*	4.032	5.162	4.067	3.831
*Standard Deviation*	1.311	1.314	1.202	1.341
*Skewness*	0.075	−0.431	−0.63	0.250
*Kurtosis*	−0.168	−0.372	−0.112	−0.462

*** *p* < 0.001.

**Table 2:** Results of the path mediation analyses.

	Estimates				Bootstrap					
								PC Confidence		
Effect	β	SE	*t*	*p*<	SE	Bias	SE-Bias	Lower	Upper	*p*
***Associations with Mediators***										
Attachment to Israel → Meaning in Life	0.23	0.028	7.96	0.0001	0.029	0.000	0.000	0.169	0.283	0.000
Attachment to Israel → Intolerance of Uncertainty	−0.12	0.027	−4.15	0.0001	0.029	0.000	0.000	−0.168	−0.054	0.000
Meaning in Life → Intolerance of Uncertainty	−0.14	0.027	−4.75	0.0001	0.028	0.001	0.000	−0.181	−0.073	0.000
***Associations with Outcome***										
Attachment to Israel → Mental Health (Direct)	0.19	0.027	7.140	0.0001	0.029	0.001	0.000	0.135	0.249	0.000
Meaning in Life → Mental Health	0.28	0.027	10.46	0.0001	0.028	0.000	0.000	0.225	0.336	0.000
Intolerance of Uncertainty → Mental Health	−0.26	0.029	−10.00	0.0001	0.032	0.001	0.000	−0.351	−0.225	0.000
***Indirect effect***					0.014			0.076	0.129	0.000

Based on 5000 bootstrap samples. PC confidence = Percentile Confidence Intervals (95%).

## Data Availability

The data file of this study is publicly available on the Open Science Framework (OSF) at: https://osf.io/8sefx/.
